# Grounded theology: a co-constructivist “two books” approach to qualitative research

**DOI:** 10.3389/fpsyg.2026.1864674

**Published:** 2026-06-17

**Authors:** Gregory J. Amundson

**Affiliations:** 1 Federal Law Enforcement Training Centers, Glynco, GA, United States; 2 Regent University School of Divinity, Virginia Beach, VA, United States

**Keywords:** grounded theology, grounded theory, qualitative research, theology and religious studies, theology (Christian), research methods

## Abstract

**Background:**

Grounded Theology (GT) is derived from the sociological research methodology Grounded Theory, originally developed by Barney Glaser and Anselm Strauss. GT aims to discover or construct theological frameworks that are firmly rooted in empirical data, achieved through systematic data collection and rigorous comparative analysis. While GT offers flexibility in its approach, it remains a complex and structured methodology. What sets GT apart is its integration of the “Two Books” theory, which posits that God reveals Himself through both the Book of Scripture (Special Revelation) and the Book of Nature (General Revelation). GT uniquely integrates insights from empirical observation of the natural world and theological reflection on scriptural texts, enabling researchers to construct frameworks grounded in both scientific and spiritual data. Successful application requires researchers to have a thorough understanding of GT’s conceptual foundations, discourse, and practical processes, as well as the interplay between these two complementary sources of revelation.

**Objective:**

To present a contemporary research framework for GT applicable to behavioral, sociological, and theological contexts. The framework supports researchers in structuring and conducting qualitative investigations that are methodologically sound and contextually relevant. The article adapts grounded theory methods through a sequenced approach involving empirical qualitative analysis, theological interpretation, and spiritual discernment. Worked examples and explanations of grounded theory concepts are provided to illustrate their relationship to GT and to ensure methodological transparency in developing rigorous, reproducible, and actionable theological frameworks.

**Results:**

The article provides a comprehensive overview of GT, supplemented by graphical and tabular representations of the processes and methods utilized in qualitative research. The framework is depicted through a diagrammatic model of the research design process, offering a clear visual guide for novice GT researchers and facilitating understanding of the methodology’s key steps and components.

**Conclusion:**

GT is a rigorous yet adaptable research framework suitable for both novice and experienced researchers interested in exploring social phenomena, particularly those grounded in qualitative data. GT research findings and recommendations have the potential to inform policy and advance knowledge, particularly in domains that address moral and ethical issues and decision-making in high-stakes environments.

## Introduction

Qualitative research represents a distinct and nuanced approach to inquiry within the broader field of research studies, offering valuable tools for understanding complex human experiences and perspectives. Within this context, the present manuscript is situated in the discipline of practical theology, with a primary focus on the methodological needs of Doctor of Ministry (D. Min.) students and Christian researchers. The proposed Grounded Theology (GT) framework is specifically designed for practitioners who operate at the intersection of theology, biblical studies, and sociological research methods. By providing a structured, reproducible approach to qualitative theological inquiry, GT enables researchers to integrate empirical findings with theological reflection in ways that are academically rigorous and contextually relevant for ministry and scholarship.

At its core, qualitative research focuses on understanding the meanings, experiences, and perspectives of individuals or groups within specific contexts (Grossoehme, 2014). Rather than focusing on generalizable findings or statistical relationships, qualitative research is interested in the contextual depth, subjectivity, and meanings that people ascribe to their experiences. This approach values rich, descriptive data over broad numerical trends, providing nuanced insights into human behavior, thoughts, and interactions that quantitative methods are not designed to elicit (Grossoehme, 2014).

## From questions to answers

Every research study begins with a clearly defined research problem, which guides the entire inquiry process. The nature of the research problem determines the philosophical assumptions that underpin the study (Birks and Mills, 2015). A philosophical perspective is a way of understanding the world that shapes the kinds of questions researchers ask and the methods they use to find answers (Mills, 2014).

Two dominant philosophical perspectives have shaped social science research. The first is positivism, which assumes that objective reality exists independently of human perception and can be known through systematic observation, measurement, and scientific methods (Taylor et al., 2016). Positivism aims to uncover objective facts and causal relationships in social phenomena, typically independent of individual subjective perspectives, and in its strongest form treats social phenomena as external “things” that act upon individuals (Durkheim, 2013).

The second perspective is phenomenology (often linked to interpretivism), which focuses on understanding social phenomena from participants’ perspectives and emphasizes how individuals perceive and experience the world, assuming that individuals define reality through personal interpretation (Taylor et al., 2016). Phenomenological research relies on qualitative methods such as participant observation and interviews, which produce descriptive data to explore lived experiences and to discover what those experiences mean to a group of people. Its central task is to understand how individuals construct their realities and make sense of their experiences, with researchers striving to see the world from participants’ perspectives (Taylor et al., 2016).

## Methodological approach

Once the philosophical perspective is established, researchers select a methodological approach that aligns with their assumptions and the research problem. Methodology refers to the theoretical and procedural framework used to investigate research questions and seek answers (Creswell, 2003). For example, a positivist perspective often leads to quantitative methodologies, while a phenomenological perspective favors qualitative methodologies.

## Research design

The methodological approach informs the development of the research design, which serves as a strategic plan to address the research questions (Birks and Mills, 2015). The research design outlines how the study will be structured, including participant selection, setting, and procedures for data collection and analysis. It ensures that the study is coherent and that each stage logically follows from the previous one.

## Methods and data collection

Within research design, researchers select specific methods for collecting and interpreting empirical evidence. Research methods are the practical tools employed within a methodological framework to generate and interpret empirical evidence (Mackenzie and Knipe, 2006). Qualitative researchers, for instance, may select interviews, focus groups, or case studies to gather rich, detailed data from study participants (Glaser and Strauss, 2017). These methods are used to gain a deeper understanding of how people construct their worldviews, make sense of their circumstances, and assign value to various aspects of their lives (Creswell, 2003).

**Figure 1:**
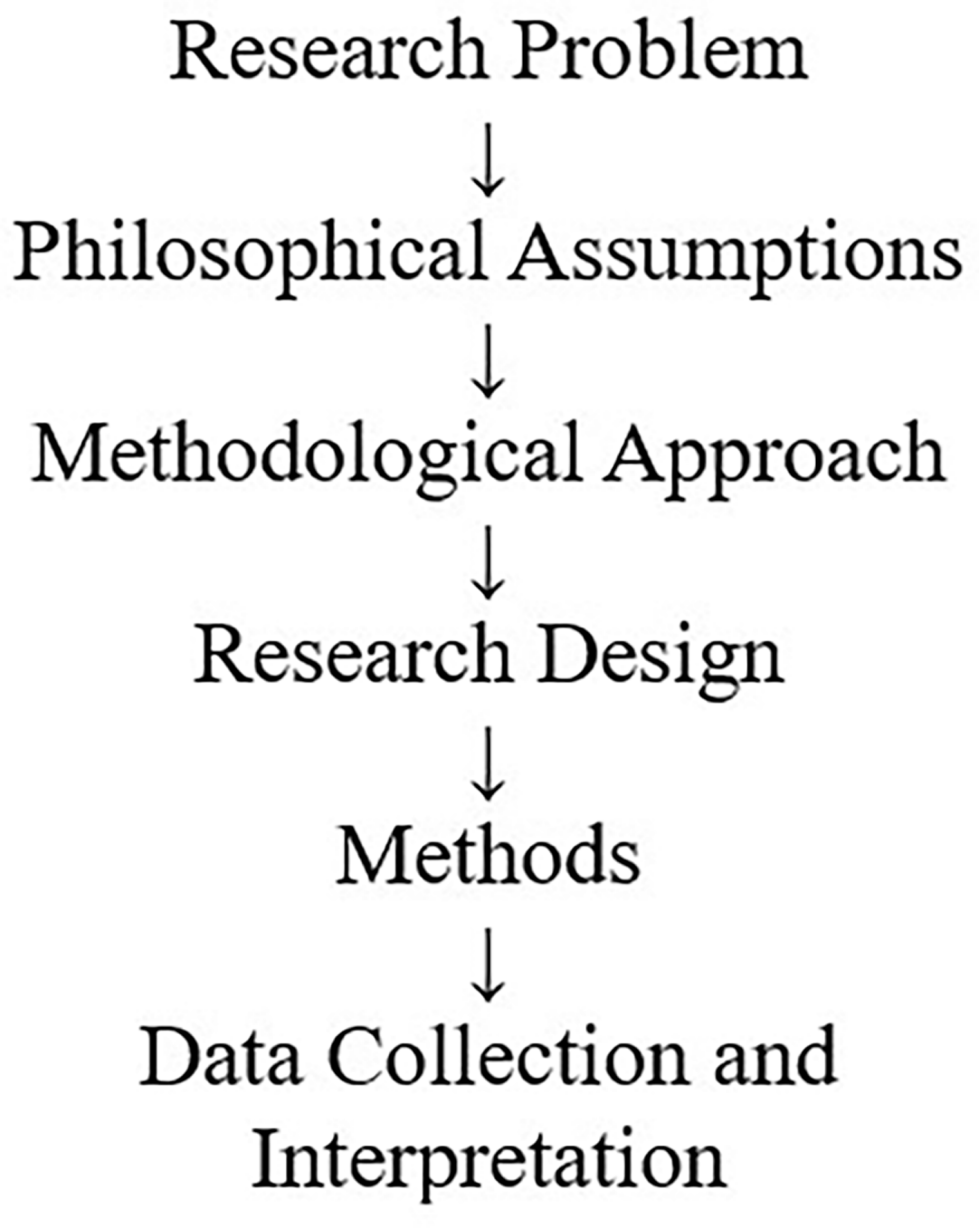
Logic flow of research process.

Figure 1 provides a visual representation that clarifies the logical sequence of the research process, illustrating how each stage builds upon the previous one and guides researchers from the initial identification of the research problem through the collection and interpretation of data (Zaiser, n.d.).

## Logic flow worked example

### Research problem

Law enforcement officers frequently encounter situations that challenge their moral values, potentially leading to moral injury (Papazoglou et al., 2020). The research problem is: How do experiences of moral injury affect law enforcement officers?

### Philosophical assumptions

To address this problem, the researcher adopts a phenomenological perspective, assuming that officers’ subjective experiences and personal interpretations are central to understanding the impact of moral injury.

### Methodological approach

Given the focus on lived experiences, the study employs a qualitative approach to explore and interpret the meanings officers assign to morally injurious events and their coping strategies.

### Research design

The research design involves selecting a purposeful sample of law enforcement officers who have reported exposure to morally challenging situations. The study is structured around in-depth, semi-structured interviews conducted in a confidential setting.

### Methods

The primary method consists of semi-structured interviews, which enable participants to describe their experiences in detail. The researcher also applies Grounded Theory (explained below) to identify patterns and themes related to moral injury.

### Data collection and interpretation

Data is collected through audio-recorded interviews, transcribed verbatim. The researcher interprets the data by coding responses and grouping them into themes such as “sources of moral injury,” “coping mechanisms,” and “factors influencing resilience.”

## Grounded theory background

Grounded theory is a “structured yet flexible research method … one of the defining characteristics of grounded theory is that it aims to *generate a theory grounded in the data*.” (Tie et al., 2019) The researchers Barney Glaser and Anselm Strauss are recognized as the founders of the grounded theory method (Glaser and Strauss, 2017). Glaser and Strauss initially collaborated in a study examining the experiences of terminally ill patients (Glaser and Strauss, 2017). During their investigation, they developed the constant comparative method, widely regarded as an innovative approach to organizing, analyzing, and coding data (Tie et al., 2019).

After concluding their study, Glaser and Strauss (1967) wrote *The Discovery of Grounded Theory*, which explained how theory could be generated inductively from data (Glaser and Strauss, 2017). Their book challenged the longstanding notion that quantitative research methods were the only scientifically valid way to make probability statements about future behavior patterns. It outlined how qualitative research could provide rigorous methods and processes to *ground theory in data* (Strauss and Corbin, 1990). Grounded theory provides researchers the means to understand and conceptualize participants’ experiences, and “knowledge gained through grounded theory methodology enables persons to explain and take action to alter, contain, and change situations.” (Strauss and Corbin, 1990).

Since its discovery, grounded theory has developed into several distinct methodological strands: traditional, evolved, and constructivist grounded theory (Tie et al., 2019). Each of these approaches builds on and extends the original work of Glaser and Strauss (Birks and Mills, 2015). Traditional or “classic” grounded theory, as Glaser described it, aims to generate a conceptual theory that explains a recurring pattern of behavior that is significant and problematic for those involved (Glaser, 1978). Evolved grounded theory is grounded in symbolic interactionism, a sociological perspective that focuses on the meanings people attach to social interactions, objects, behaviors, and events based on what they believe to be true (Griffin, 2006). Constructivist grounded theory (also working from a symbolic interactionist tradition), is rooted in constructivism and emphasizes how participants construct meaning in relation to the topic under study, with the researcher actively co-constructing experiences and interpretations alongside them (Charmaz, 2014).

## Patristic foundations of grounded theology

Biblical interpretation has been a central component of the Church’s theological development throughout Christian history (Duesing and Finn, 2021). From the Apostolic period to the early medieval era, the Church Fathers employed diverse exegetical methods to draw theological meaning from Scripture, often integrating philosophical reasoning, historical context, and lived experience (Allison, 2011). Their interpretive frameworks laid the foundation for Christian theology, shaping doctrinal development, pastoral theology, and apologetics. GT shares key methodological similarities with the patristic approach to biblical interpretation, particularly in its use of systematic analysis, theological synthesis, and engagement with Scripture and lived experience. By examining the interpretative traditions of the Church Fathers, we can trace the historical continuity between ancient theological inquiry and GT.

The early Church Fathers developed four primary methods of biblical interpretation, which became foundational for Christian theology: (Allison, 2011) The literal, or historical, interpretation sought to uncover the plain meaning of the biblical text, often considering its historical and cultural context. Irenaeus of Lyons (c. 130–202 AD) was a prominent advocate of this approach, particularly in his defense against Gnostic interpretations that distorted biblical meaning. His work, *Against Heresies*, demonstrated the necessity of grounding theology in the historical and textual reality of Scripture.

Alongside the literal approach, allegorical interpretation emerged, especially within Alexandrian theology. Figures such as Origen (c. 185–254 AD) argued that Scripture contained deeper, hidden meanings beyond the literal text. Origen’s *Hexapla* and his spiritual exegesis of the Old Testament introduced a layered understanding of biblical meaning, correlating historical events with Christological fulfillment and spiritual insight.

Typological interpretation was also central to the work of many Church Fathers, including Augustine of Hippo (354–430 AD) and Cyril of Jerusalem (c. 313–386 AD). This method emphasized the way Old Testament figures and events prefigured New Testament realities, linking empirical history with theological significance. Typology aligns with grounded theology’s harmonization process, in which emerging empirical and scriptural themes are analyzed together to reveal deeper connections.

Finally, moral (tropological) and anagogical interpretations were promoted by Fathers such as Gregory of Nyssa (c. 335–395 AD) and John Cassian (c. 360–435 AD). These approaches focused on the ethical and eschatological application of Scripture, viewing its truths as spiritually transformative and functionally relevant. This principle resonates with GT’s emphasis on practical theology, where theological insights are intended to shape both personal character and practical ministry.

The Church Fathers’ hermeneutical approach shares several methodological commonalities with GT, particularly in how theological insights emerge from textual engagement and contextual experience. The use of the Septuagint, intertextual analysis, and early theological commentaries demonstrates a methodologically structured approach that mirrors the systematic coding and synthesis of GT.

Many Church Fathers integrated lived human experience, philosophy, and historical realities into their theological interpretations. For example, Augustine’s *Confessions* combined autobiographical reflection with scriptural theology, much like GT’s integration of personal narratives in theological research (Duesing and Finn, 2021). Athanasius of Alexandria’s (c. 296–373 AD) defense of Christ’s Divinity in *On the Incarnation* employed empirical reasoning to demonstrate theological truths, paralleling the use of General Revelation in GT’s theological inquiry (Duesing and Finn, 2021). GT reflects this patristic tradition by integrating Scripture with sociological, behavioral, psychological, and historical data while maintaining doctrinal fidelity through theological harmonization and synthesis.

The Fathers saw theology as a living discipline. Their pastoral letters, doctrinal formulations, and liturgical reflections show how theology was intended to shape worship, ethics, and community life (Allison, 2011). GT adheres to this principle, emphasizing that theology must be both academically rigorous and functionally applicable. The Council of Nicaea (325 AD) exemplifies this approach, where biblical exegesis, lived ecclesial realities, and doctrinal synthesis converged to form the Nicene Creed, an example of theological harmonization leading to practical theology (Duesing and Finn, 2021).

The Church Fathers’ structured approach to research and theological development is reflected in the methodology of GT. By integrating theological sampling, empirical engagement, and rigorous methodological coding, grounded theologians combine qualitative research, theological reflection, and systematic analysis to develop functional and biblically grounded theology. Ultimately, GT advances the early Christian tradition by ensuring that theology actively engages with God’s Word, Creation, and the realities of human experience.

## Distinctions from traditional grounded theory

The origins of grounded theory were rooted in post-positivism and objectivism (Burns et al., 2022). It sought to systematically discover theory from data, assuming researchers could objectively observe reality through inductive coding and constant comparison. The founders of grounded theory emphasized that researchers should avoid imposing preconceived ideas on the data (Burns et al., 2022). They maintained that grounded theory was a neutral and empirical method for identifying patterns already present in the data rather than constructing meaning through interpretation.

The original framework for grounded theory assumed that “the data speaks for itself,” (Reay et al., 2016) indicating that meaning emerges naturally from systematic analysis. However, GT maintains that only God’s Word can truly speak for itself. While GT engages with data from Special and General Revelation through rigorous research, we recognize that theological meaning is guided by the Holy Spirit and not merely derived from empirical patterns. Unlike grounded theory’s objectivist and postpositivist stance, GT follows a constructivist approach, recognizing that meaning is “co-constructed”—not only between the researcher and the data but also through the active work of the Holy Spirit.

While traditional grounded theory assumes that theory “emerges” from data in a neutral and systematic manner, GT acknowledges that the Holy Spirit shapes human interpretation and contextual influences. This approach aligns with the biblical understanding that God’s truth is revealed through Scripture and human experience, requiring the researcher to engage in an interpretive process that integrates Special and General Revelation. Unlike grounded theories, which have a foundational insistence on an objective, empirical discovery of patterns already present in the data, GT holds that theological insights emerge through a structured coding process in which the Holy Spirit actively works with the researcher to uncover deeper realities. This co-constructivist stance does not negate the importance of systematic analysis. Instead, it acknowledges that GT develops meaning and theory through engagement with Special and General Revelation, as well as established empirical research methods.

## Clarifying the epistemological foundations of GT

Grounded Theology (GT) is intentionally positioned at the intersection of constructivism, realism, and theological epistemology, seeking to bridge the gap between empirical inquiry and confessional theological commitments. The epistemological stance of GT can be described as “co-constructivist,” yet it is distinct from secular constructivism in important ways.

In traditional constructivist grounded theory, knowledge is understood as being co-constructed by researchers and participants through their situated interactions and interpretations. GT affirms the value of meaning-making and recognizes that researchers bring their own perspectives, experiences, and theological backgrounds to the interpretive process. In this sense, GT is constructivist: it acknowledges that theological understanding is shaped through dialogue with empirical data, lived experience, and the interpretive community.

However, GT also affirms a form of theological realism rooted in the conviction that Divine Revelation, encompassing both Special Revelation (Scripture) and General Revelation (Creation), communicates objective truth about God and reality. While human interpretations are necessarily limited and situated, GT maintains that theological inquiry is ultimately oriented toward truth that exists independently of the researcher’s perspective. This commitment to realism is grounded in the authority of Scripture as the primary source of theological data, interpreted in light of the broader Christian tradition and the witness of creation.

The “co-constructivist” stance in GT thus refers to the dynamic interplay between empirical inquiry, spiritual discernment, and theological reflection. Theological truth is discerned not solely through human reasoning or social construction, but through a process in which the researcher actively engages with empirical data, interprets it in dialogue with Scripture, and remains open to the illuminating work of the Holy Spirit. In this paradigm, the Holy Spirit is understood as an active co-constructive agent, guiding both the interpretive process and the formation of theological theory. This approach is particularly relevant for practical theology, where the integration of lived experience, biblical interpretation, and spiritual discernment is essential for developing theological frameworks that are both contextually meaningful and faithful to Christian doctrine.

By explicitly articulating this epistemological foundation, GT provides a transparent framework for integrating empirical and theological sources of knowledge. It acknowledges the complexity of theological meaning-making, the authority of Divine Revelation, and the necessity of reflexivity and humility in the interpretive process. In doing so, GT offers a distinctive contribution to practical theology and qualitative research, equipping practitioners to navigate the challenges of interdisciplinary inquiry while remaining rooted in confessional commitments.

## Scholarly context and rationale for GT

Grounded Theology (GT) builds upon and extends a rich tradition of scholarship in practical theology, theological ethnography, lived religion, interpretive sociology, and qualitative religious research. Recent decades have seen increasing interest in methodologies that integrate empirical inquiry with theological reflection, particularly within the fields of practical theology and qualitative religious studies.

Many Christian researchers and D. Min. students frequently reach for sociological research methods in their studies, recognizing the value of empirical approaches for understanding lived experience and social dynamics. At the same time, they often yearn to incorporate their years of theological and biblical study into their research design, seeking ways to honor both their academic training and their confessional commitments. In addition, these researchers desire to explain, document, and substantiate the guidance of the Holy Spirit throughout the research process, ensuring that spiritual discernment is integrated with methodological rigor. GT offers a way of bridging these two worlds, providing a methodological framework that integrates empirical inquiry with theological and biblical reflection.

Practical theology has long emphasized the importance of connecting theological concepts to lived experience and ministry contexts. John Swinton’s *Practical Theology and Qualitative Research* (2016) provides a foundational framework for integrating qualitative methods with theological reflection (Swinton, 2016). Swinton argues that practical theology must attend to the complexities of human experience, using empirical research to inform and refine theological understanding. GT shares this commitment, offering a structured approach to bridging empirical findings and theological interpretation.

Theological ethnography and the study of lived religion have further expanded the scope of qualitative inquiry in theology. Tanya Luhrmann’s *When God Talks Back* (2012) explores how religious communities experience and interpret Divine communication, employing ethnographic methods to examine the intersection of belief, practice, and everyday life (Luhrmann, 2012). Robert Orsi’s *Between Heaven and Earth* (2013) investigates the ways in which religious experience is shaped by social, cultural, and historical contexts, emphasizing the interpretive complexity of lived religion (Orsi, 2013). These works highlight the importance of attending to both subjective experience and broader theological frameworks, a principle central to GT. Despite these advancements in practical theology and qualitative religious research, there remains a need for a more unified and systematic approach that fully integrates empirical inquiry with theological and biblical reflection.

## Positioning GT within theological studies

Grounded Theology (GT) aligns most closely with practical theology due to its shared emphasis on real-world application and lived experiences. Both approaches prioritize the relational and experiential aspects of faith, recognizing that theological concepts must be grounded in the everyday realities of human life. Both disciplines seek to integrate theory with practice by exploring how biblical truths are embodied in tangible actions and lived experiences. While practical theology often engages directly with the social, cultural, and communal aspects of faith, GT brings a deeper focus on Scripture as the ultimate source of Revelation, interpreting human experience and societal issues through the lens of biblical teaching (Muller, 2013). Despite their similarities, GT maintains a more systematic and text-centered methodology. In contrast, practical theology may draw more freely from interdisciplinary fields, such as the social sciences and narrative studies. Nonetheless, both fields aim to bridge the gap between theological knowledge and its practical implications in the real world, making them highly complementary in the modern theological landscape.

The parallels between GT and practical theology become especially clear when considering how each discipline uses narrative as a primary tool for understanding. Practical theology’s use of narrative metaphor and narratology, which involves analyzing and interpreting stories, mirrors GT’s emphasis on the lived experience of individuals as a source of theological insight (Muller, 2013). Both disciplines seek to uncover the deeper meanings embedded within human stories and social practices, allowing researchers to draw connections between the experiences of individuals and broader theological truths. In this way, both disciplines embrace a holistic view of human experience, recognizing that theological reflection must be rooted in individuals’ context and lived realities.

Additionally, GT and practical theology strive to break down disciplinary boundaries, fostering a broader dialogue between theology, the social sciences, and other fields. Practical theology recognizes the value of engaging with various academic disciplines, ranging from the social sciences to the natural sciences, to deepen its understanding of human society and the religious community (Muller, 2013). This interdisciplinary approach aligns closely with GT’s focus on integrating diverse insights from Scripture and General Revelation to address contemporary issues. Just as practical theology aims to open boundaries between various research fields to gain a fuller understanding of the human condition, GT also encourages an exchange of knowledge that transcends traditional disciplinary limits, offering a more nuanced and comprehensive view of Special Revelation and human experience.

Despite their similarities, there are key areas where GT departs from practical theology, especially in its systematic coding and approach to Scripture.

1) *Primary source of authority*: One of the most significant differences between GT and practical theology lies in the role and emphasis placed on Scripture. GT views Scripture as the ultimate and primary source of data and authority, considering it the “pure and raw material” from which all theological insights should emerge. In contrast, while practical theology uses Scripture as a foundational resource, it often prioritizes lived experience, narrative, and empirical data gathered from human situations and social sciences. GT systematically interprets Scripture through a lens that integrates human experience but always returns to Scripture as the definitive guide for understanding God and human behavior. On the other hand, practical theology may engage more directly with personal testimonies, cultural narratives, and contemporary societal issues, allowing those to shape the theological reflection more directly.
2) *Theological methodology*: GT is a methodologically rigorous approach, employing both qualitative and quantitative methods. However, it remains firmly anchored in Scripture, using the Bible to interpret General Revelation and human experience. In this sense, GT can be seen as more systematic and prescriptive, relying on Scripture as the final arbiter of truth while considering other forms of revelation as secondary. In contrast, practical theology often operates within a more flexible, context-driven framework, adapting its methods to address the specific needs of the community or social issue at hand (Muller, 2013). This flexibility enables practical theology to prioritize individuals’ lived experiences and stories, often integrating interdisciplinary insights without relying solely on Scripture as the authority.
3) *Focus on relational knowing*: GT emphasizes relational knowing, highlighting that knowing God is not just intellectual assent but an active, personal relationship with Him. Central to this approach is the belief that knowing God involves engaging deeply with His Word, experiencing the transformative power of the Holy Spirit, and living out faith in everyday life. While GT’s focus on relational knowing centers on an ongoing engagement with Scripture, practical theology may be more concerned with immediate action and engagement within specific contexts, sometimes prioritizing context over doctrine.
4) *Transcendence vs. contextualization*: GT maintains a more transcendent and universal approach to truth, asserting that Scripture provides the primary lens through which we interpret all human experience. Practical theology, conversely, is more contextual, focusing on how theology meets the immediate needs of specific communities, cultures, and social structures (Muller, 2013). This emphasizes practical engagement with societal issues, where theological reflection may shift depending on local, cultural, or social circumstances. While GT remains firmly tied to Special Revelation, practical theology often takes a more fluid and adaptive approach, allowing for the contextualization of theological insights.

## Towards a unified and systematic approach

The idea of GT has been explored in various theological and academic contexts; however, existing studies have not fully developed a structured, systematic methodology that integrates Special Revelation (Scripture) and General Revelation (Creation) into a cohesive theological framework (Amundson, 2026). While scholars have applied aspects of grounded theory to theology, their approaches often remain fragmented, subjective, or limited to specific contexts without providing a unified system for theological development.

In *Ethnography as Christian Theology and Ethics* (2011), Christian Scharen and Aana Marie Vigen integrate ethnographic methods into theological research, emphasizing the importance of lived experiences within communities (Scharen and Vigen, 2011). Their work highlights contextual theology and grounded theory but lacks a structured process for systematically synthesizing these experiences into a coherent theological framework (Scharen and Vigen, 2011). In exploring “grounded theologies,” Justin K. H. Tse (2014) examines how religious and secular practices shape human geography (Tse, 2014). He argues that these practices can be viewed as performative acts of place-making informed by conceptions of the transcendent (Tse, 2014). Tse’s notion of grounded theologies concerns how theological ideas, whether religious or secular, shape the spatial, social, and political dimensions of human life.

Tse challenges the rigid division between the religious and the secular, positing that religious and non-religious worldviews operate as theologies, engaging with the transcendent and influencing cultural place-making. He emphasizes how theological assumptions shape the world, even in secular contexts. However, Tse’s framework lacks a systematic method for evaluating theological claims. The author’s approach to GT transcends descriptive cultural analysis; it offers a structured, rigorous framework for developing systematic and functional theology by integrating biblical exegesis, empirical observation, and structured coding methods.

In discussing his work on grounded theology, Bruce A. Stevens (2016) notes its limitations, stating:

*It seems unlikely that grounded theology will ever deliver a coherent, systematic theology*. It does not provide a methodology for moving from many perspectives to one viewpoint. *There is a stopping point for personal beliefs*. It explores the credo or ‘I believe.’ Perhaps it is a methodology that can be applied to messy concepts such as luck. It is well-suited to the discipline of practical theology—it has a low center of gravity. It is grounded (Stevens, 2016).

Stevens effectively applies grounded theory to theological inquiry, but his approach remains limited to personal theological exploration rather than providing a pathway toward a coherent, structured, and functional systematic theology. His acknowledgment that grounded theology has a “low center of gravity” and serves as a “stopping point for personal beliefs” suggests that prior approaches have lacked the methodological rigor necessary to develop widely applicable theological frameworks.

In contrast, the author’s approach to GT introduces a rigorous and systematic methodology that moves beyond individual belief structures toward academically sound, functional theological frameworks (Amundson, 2026). Academic rigor is developed through a structured coding process, allowing theological insights to be methodically designed, tested, and refined. Unlike previous approaches, the method presented herein provides a clear path from data collection to systematic theological formulation, ensuring that grounded theology is a scientific, reproducible, and academically rigorous discipline (Table 1).

**Table 1:** Advancements in grounded theology.

Grounded theology (Author’s method)	Advancements to other approaches
Methodology	A structured, systematic, and iterative approach using rigorous coding and analysis techniques.
Theological framework	Integrates Special Revelation (Scripture) and General Revelation (Creation) to build a coherent, reproducible theology.
Coding process	A structured coding process (Illumination, Harmonization, Synthesis, Summation) identifies and validates theological patterns.
Cognitive bias mitigation	Employs strategies to recognize and counteract bias, ensuring objective theological conclusions.
Application scope	Designed for academic, governmental, judicial, pastoral, and hermeneutical contexts.
Engagement with revelation	Views both Scripture and Creation as authoritative sources that must be studied in harmony.
Systematic theology	Provides a pathway to constructing coherent, functional theological frameworks.

## Fundamentals of GT

Grounded Theology (GT) is a functional research paradigm that integrates empirical inquiry, theological reflection, and Divine guidance to develop *theological theory* that is both academically rigorous and practically applicable. In this context, “theological theory” refers to conceptual frameworks and mental models developed through the integration of empirical research, theological reflection, and biblical interpretation. Rooted in the “Two Books” of Special Revelation (Scripture) and General Revelation (Creation), it operates from the conviction that God reveals Himself coherently through both His written Word and the natural world (Ross, 2018). Drawing on Hugh Ross’s (2017) articulation of Dual Revelation, GT affirms that any genuine conflict does not arise from Revelation itself but from human misinterpretation, whether of Scripture, of nature, or both (Ross, 2018). Thus, while “all is data,” (Glaser, 2002) Scripture, specifically the 66 canonical books, remains the primary and authoritative data source, and all other sources (historical, ecclesial, academic, scientific, experiential) are interpreted in light of biblical truth.

Within this framework, Scripture functions as theological data at multiple levels: individual words and phrases serve as unstructured data, sentences as semi-structured data, and larger units such as narratives, parables, sermons, and speeches as structured data. General Revelation, including scientific findings, historical records, and lived experience, provides secondary data that can illuminate and triangulate theological insights, so long as it is consistently read through a biblical lens. In this way, GT both honors the dictum in grounded theory that “all is data” (Glaser, 1998) and upholds the theological conviction that “all Scripture is God-breathed,” (*2 Timothy* 3:16–17, n.d.) treating the biblical text as living, active, and definitively authoritative for faith and practice.

## Clarifying methodological and theological claims

Grounded Theology (GT) intentionally distinguishes between confessional commitments and methodological procedures to ensure transparency and rigor within interdisciplinary research contexts. Confessional commitments, including the belief in the Holy Spirit’s guidance, are acknowledged as foundational to the theological perspective of the researcher. These commitments inform the interpretive lens through which empirical data and Scripture are engaged, but they are not themselves methodological steps. Instead, GT treats spiritual discernment and theological conviction as contextual factors that shape, but do not dictate, the research process.

Within GT, theological claims such as the authority of Scripture and the significance of Special Revelation are treated as sources of data and interpretive frameworks. Methodologically, GT employs established qualitative research procedures, including purposive sampling, constant comparison, and a structured coding sequence consisting of illumination, harmonization, synthesis, and summation. These procedures are applied to both empirical and theological data. The GT process is intentionally designed to be transparent and reproducible, enabling researchers from various confessional backgrounds to follow the same methodological steps.

To facilitate interdisciplinary evaluation, GT encourages explicit reflexivity. Researchers are required to document their assumptions, theological commitments, and interpretive decisions throughout the research process. This reflexive practice enables readers from diverse backgrounds to understand how theological claims influence interpretation and to critically assess the findings.

GT also employs triangulation and engagement with broader scholarship as mechanisms for adjudicating competing theological interpretations and avoiding circularity. Triangulation involves cross-referencing emerging theological insights with empirical findings, established doctrinal sources, and relevant academic literature. When alternative interpretations or contradictory findings arise, GT requires researchers to address these explicitly, compare them with established theological and empirical evidence, and articulate the rationale for their conclusions.

By integrating reflexivity, triangulation, and scholarly engagement, GT provides a methodological structure that supports rigorous evaluation of theological claims within qualitative research. This approach ensures that theological interpretations are not simply asserted, but are critically examined, validated, and situated within the wider context of practical theology and interdisciplinary inquiry.

## Sequencing empirical and theological inquiry

Although GT integrates empirical inquiry, theological interpretation, and spiritual discernment within a unified research paradigm, these components can be analytically distinguished and described as a staggered process. This clarification preserves the integrity of empirical qualitative methods while maintaining the theological aims of the approach (Zaiser, n.d.).

First, empirical data from lived experience and observation are collected and analyzed using established grounded theory procedures. At this stage, researchers treat narrative accounts, behavioral observations, and other forms of General Revelation as qualitative data. They apply constant comparison, coding, and category development to identify patterns and processes that emerge from the data itself, without initially imposing theological categories.

Second, the emergent empirical findings are interpreted through theological and hermeneutical frameworks. In this phase, the categories developed through grounded theory are brought into dialogue with Scripture, historical theology, and established doctrinal resources. The purpose is to examine how the empirically grounded categories resonate with, challenge, or are refined by the biblical witness and the broader theological tradition.

Third, theological reflection, including explicit engagement with Special Revelation and spiritual discernment, functions as a higher-order interpretive lens. Here, the researcher seeks to articulate a theological theory that is both empirically informed and biblically faithful, recognizing the Holy Spirit’s role in illuminating Scripture and guiding understanding. The resulting theological constructs, refined through the GT coding process, are thus grounded in empirical data, tested against Scripture and tradition, and framed within a coherent theological epistemology.

By making this sequence explicit, GT maintains methodological transparency for interdisciplinary audiences. Empirical qualitative methods remain intact and recognizable, while theological and spiritual dimensions engage the findings in a structured, progressive manner.

## Sampling and theological sensitivity

Following the process developed by Glaser and Strauss (1967), GT researchers begin by deliberately selecting data sources most likely to yield valuable insights relevant to their research question (Glaser and Strauss, 2017). This approach, known in grounded theory as purposive sampling, ensures that the initial data collected is relevant and informative in building the study’s foundation (Glaser and Strauss, 2017). As data is collected, coded, and analyzed, the initial sample forms the foundation for further investigation. GT researchers then use theological sampling, choosing new sources based on emerging themes, gaps, or questions identified during initial analysis. Theological sampling follows clues in the data to deepen, clarify, and saturate developing categories, ensuring that the final theory is firmly rooted in the data and evolves as new insights emerge. This process is akin to a physician diagnosing a patient: as initial symptoms and test results are analyzed, the physician pursues additional tests or consults further sources to clarify uncertainties, fill gaps, and refine their understanding. Each new piece of information helps deepen and saturate the diagnostic categories, ultimately leading to a well-grounded, evolving diagnosis.

Throughout the research process, theological sensitivity is crucial. Building on the concept of theoretical sensitivity introduced by Glaser and Strauss (1967), theological sensitivity refers to the researcher’s ability to discern which segments of data are significant for developing theological theory (Glaser and Strauss, 2017). Strauss and Corbin (1990) describe theoretical sensitivity as insight into what is meaningful and important in the data, while Birks and Mills (2015) define it as the skill of identifying and extracting elements relevant to the emerging theory (Birks and Mills, 2015).

In GT, theological sensitivity is a foundational principle that enables researchers to code and interpret Scripture and theological phenomena with depth, discernment, and fidelity to Divine Truth. This process requires researchers to recognize their own biases and assumptions, and to understand that theological insight is co-constructed through the interplay of the Holy Spirit, Special Revelation, lived experience, and academic inquiry. Theological sensitivity is cultivated through iterative research methods such as memo-writing, constant comparison, and coding, which enable insights that remain faithful to biblical truth while incorporating contemporary perspectives. GT researchers are encouraged to examine Scripture with nuance, attending to linguistic, historical, and contextual factors, and integrating their professional, cultural, and spiritual experiences. By maintaining humility and openness to the Holy Spirit, GT researchers strive to develop rigorous, applicable, and transformative theological frameworks that uphold the integrity of Special Revelation.

## Overview of grounded theology coding

Methodologically, GT adapts grounded theory’s constant comparative logic and coding procedures to theological inquiry, with Scripture as the primary source and the Holy Spirit as an active co-constructive agent. It employs a structured coding sequence designed to move from raw data to functional theology: Illumination Coding involves “fracturing” (Birks and Mills, 2015) data from Scripture and Creation to surface initial themes and patterns; Theological Sampling uses cross-referencing, hermeneutical comparison, and literary parallels to ensure that emerging insights are exegetically sound rather than driven by subjective presuppositions. Harmonization Coding then integrates these themes into coherent theological categories and tests their consistency across the canon and relevant empirical or historical data. Synthesis Coding refines these categories into higher-order theological constructs, with the researcher carefully journaling decisions to maintain transparency and rigor. Finally, Summation Coding distills the resulting data into a cohesive, communicable, and practically applicable theological framework, transforming multilayered constructs into usable, functional theology.

Throughout this process, GT maintains a co-constructivist stance in which the Holy Spirit actively shapes how patterns are discerned, data are synthesized, and theories are developed. Theological knowledge is therefore not solely the product of human reasoning or empirical observation but is spiritually discerned and divinely illuminated, as Scripture affirms: “We have received not the spirit of the world, but the Spirit who is from God, that we might understand the things freely given us by God… not taught by human wisdom but taught by the Spirit, interpreting spiritual truths to those who are spiritual.” (*1 Corinthians:2 12–13,* n.d.) In this sense, GT is both empirically engaged and theologically faithful, framing research as an act of partnership with God and a form of worship, where disciplined qualitative research methods, rigorous exegesis, and practical ministry application are brought together in a unified, Spirit-led inquiry into Divine Truth.

**Figure 2:**
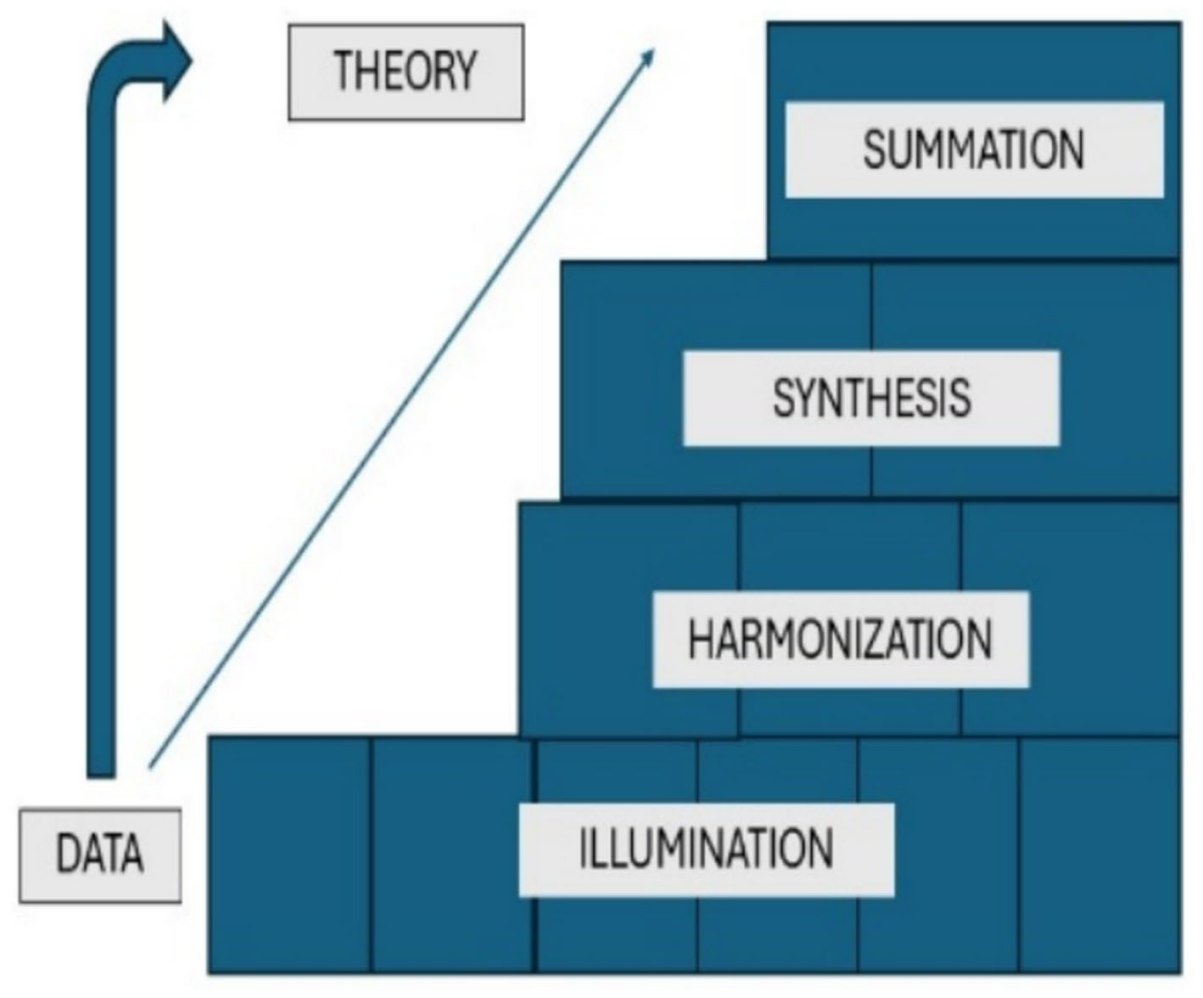
From fractured data to coherent theory.

Figure 2 illustrates the iterative and progressive coding process in GT, in which data is initially fractured at the illumination stage, where discrete words, phrases, or empirical observations are identified, and then systematically incorporated into increasingly abstract levels of analysis. Through harmonization coding, these fragmented data points are grouped into broader conceptual categories; synthesis coding further refines and interrelates these categories into higher-order theological principles; and summation coding ultimately consolidates these insights into a cohesive, actionable theoretical framework. The figure visually represents how raw data is progressively elevated through each coding phase, ensuring that the final theory is firmly grounded in both the empirical and theological dimensions of the research.

## Data collection in general revelation

When properly understood and analyzed, General Revelation and natural science can be the most fruitful sources of data for GT researchers (Richards et al., 2021). Natural science seeks to systematically understand the universe by following the evidence wherever it leads, uncovering events, rules, principles, concepts, and laws that explain phenomena (Richards et al., 2021). It involves an ongoing feedback loop between hypotheses and emerging theories related to General Revelation. In some instances, GT researchers can apply the principles of natural science in controlled field experiments that are replicable and testable by other researchers. In other cases, researchers must carefully and systematically observe social settings to gain a deeper understanding of human behavior and interactions. Additionally, science may involve piecing together clues to explain past events and determine which hypotheses best account for them (Richards et al., 2021). Through these approaches, natural science becomes a vital tool for GT researchers in their pursuit of understanding natural phenomena within God’s creation.

## Fine-tuning and analytical reasoning

General Revelation and natural science align with the concept of fine-tuning. Fine-tuning refers to the specific conditions required for the existence of complex life in the universe (Moreland and Craig, 2017). These conditions encompass fundamental characteristics, such as the laws of nature, their numerical constants, and the particular attributes that planets require to support life (Richards et al., 2021). The core idea is that these factors must fall within an extraordinarily narrow range for chemical-based life to be viable. This delicate balance suggests that the universe possesses intrinsic value and purpose. Within the framework of General Revelation, fine-tuning provides compelling evidence that scientific discoveries may reveal the intentional design of a Creator, serving as a powerful argument for the existence of God in apologetics.

General Revelation invites the application of *logic*. Logic examines the principles of sound reasoning and focuses on *when*, *how*, and *why* we can draw legitimate conclusions from premises (Moreland and Craig, 2017). Three types of logic (deductive, inductive, and abductive) are central to the reasoning and development of theological theories within the context of General Revelation.

Deductive reasoning moves from general and known truths to a specific conclusion (Richards et al., 2021). For example, if A is greater than B, and B is greater than C, then A must be greater than C (Holden et al., 2021). Mathematics and philosophical reasoning typically employ this form of logic. Provided that the premises are accurate and the structure of the argument is valid, deductive logic produces certain conclusions. Those who believe in God often reason deductively, moving from effects to their ultimate cause, from “codes to Coder, creation to Creator, from the dependent to the Independent, from design to Designer, from information to Informer, from law to Legislator, from meaning to Meaner, from mind to Mind.” (Holden et al., 2021).

Proverbs 24:30–34 offers an insightful example of how General Revelation can lead to more specific, deductive conclusions, illustrating the transition from observing nature to moral and theological insights.

I went past the field of the sluggard,

past the vineyard of someone who has no sense;

thorns had come up everywhere,

the ground was covered with weeds,

and the stone wall was in ruins.

I applied my heart to what I observed

and learned a lesson from what I saw:

A little sleep, a little slumber,

a little folding of the hands to rest—

and poverty will come on you like a thief

and scarcity like an armed man (*Proverbs* 24:30–34, n.d.).

In this passage, the author reflects on a neglected field overtaken by weeds, using this natural observation as a metaphor for a lazy, irresponsible life. The sight of the unkempt field serves as General Revelation, a visible sign of disorder and decay that is universally observable in the natural world. The weeds themselves, representing neglect and inaction, are not an isolated observation but a broader, symbolic truth that connects the physical state of the world to human behavior. Observing nature allows the biblical author to make a higher, Holy Spirit-inspired deductive conclusion: laziness and poor stewardship lead to ruin in physical environments and human lives.

The passage thus exemplifies how General Revelation, such as the state of a field, provides a foundation for GT researchers to draw more specific, moral conclusions about human conduct. By noticing the effects of neglect in nature, the author logically deduces that the same principle applies to human laziness: just as a neglected field becomes overrun with weeds, a lazy person’s life will inevitably become overrun with poverty and hardship. The movement from General Revelation, in the form of observable natural phenomena (such as weeds), to a specific moral conclusion (the consequences of laziness) reflects a higher, deductive process of reasoning. It underscores the relationship between natural observation and the theological lessons it can yield, demonstrating how the natural world, as part of God’s creation, informs our understanding of human behavior and ethical living.

Conversely, inductive reasoning involves drawing general truths from specific facts and data. Scientific research, including experiments, is commonly inductive, drawing conclusions from observed patterns or empirical data (Holden et al., 2021). The author previously utilized the analogy of a physician diagnosing a patient to illustrate the iterative process of sampling and analysis; in this section, the analogy of a law enforcement detective is employed to underscore how researchers follow emerging evidence to refine theoretical conclusions. An example of inductive reasoning in the Bible is found in Proverbs 6:6–8, where the author observes the behavior of ants to teach a broader lesson about diligence and laziness. Inductive reasoning involves drawing general conclusions based on specific observations.

Go to the ant, you sluggard;

consider its ways and be wise!

It has no commander,

no overseer or ruler,

yet it stores its provisions in summer

and gathers its food at harvest (*Proverbs* 6:6–8, n.d.).

The author uses the specific, observable behavior of ants to make a broader point about the importance of hard work and planning. The ant works diligently without a supervisor, gathering food during the proper seasons, which serves as a model for the individual to avoid laziness. By observing the ants’ actions, one can conclude that the value of being industrious and not idle leads to a general principle about work and responsibility. This passage is a classic example of inductive reasoning: specific observations (the ant’s behavior) lead to a general conclusion (the need for diligence and the dangers of laziness).

Finally, abductive reasoning processes ideas from our subconscious minds, which surface as flashes of insight or intuition in our conscious thoughts (Holden et al., 2021). Those experiencing these insights may be unable to articulate how they arrived at the idea; yet, abductive reasoning can lead to valuable hypotheses and a deeper understanding (Richards et al., 2021).

## Abductive reasoning and the Holy Spirit

Matthew 16:13–17 illustrates abductive reasoning. In this passage, Peter’s declaration is not based on formal logic or systematic reasoning but rather on a moment of insight, which Jesus attributes to Special Revelation through the Holy Spirit.

When Jesus came to the region of Caesarea Philippi, he asked his disciples, “Who do people say the Son of Man is?”

They replied, “Some say John the Baptist; others say Elijah; and still others, Jeremiah or one of the prophets.”

“But what about you?” he asked. “Who do you say I am?”

Simon Peter replied, “You are the Messiah, the Son of the living God.”

Jesus replied, “Blessed are you, Simon son of Jonah, for this was not revealed to you by flesh and blood, but by my Father in heaven.”

In this instance, Peter’s insight comes from a profound, intuitive understanding that Jesus is the Messiah. Jesus affirms that this Special Revelation was not something Peter reasoned out by human logic (“not revealed to you by flesh and blood”) but rather something revealed to him by God through the Holy Spirit. The abduction process often transcends explicit reasoning or evidence, relying instead on a sudden, intuitive understanding or insight—what we might recognize as a flash of Divine insight. This example aligns with how abductive reasoning often surfaces in our consciousness as an instinctive or inspired conclusion, in this case, through the Holy Spirit, who guides Peter to recognize the truth of who Jesus is.

The Holy Spirit guides GT researchers during their study, providing insight and understanding that may not be immediately accessible through conscious reasoning alone. Furthermore, the Holy Spirit is essential in aiding abductive reasoning and helping the researcher navigate the methodological process with clarity and spiritual discernment. Abductive reasoning often involves sudden bursts of clarity or intuitive glimpses into natural phenomena, which, within the context of theological sensitivity, may be illuminated by the Holy Spirit to clarify Scripture or General Revelation.

This premise is reflected in passages such as James 1:5, where Scripture promises: “If any of you lacks wisdom, let him ask of God, who gives generously to all without finding fault, and it will be given to him.” Similarly, 1 Corinthians 2:10–12 reveals that the Holy Spirit searches the depths of God, revealing to believers what no human could know on their own (*Additional Passages include Luke 12:12, John 14:26, Romans 8:16, and 1 John* 2:27, n.d.). These verses emphasize that the Holy Spirit is indispensable for guiding the GT researcher into a deeper understanding of God’s Truth, enabling them to comprehend spiritual wisdom that surpasses human reasoning. In this way, the Holy Spirit can enhance a GT researcher’s ability to perceive deeper meanings and connections that align with God’s Dual Revelation, enabling them to form theological theories that faithfully resonate with Scripture and the natural world.

## Data collection in special revelation

While the Bible serves as God’s Revelation for all humanity, its authors initially addressed it to their original audience before it was later contextualized in modern interpretations (Keathley et al., 2017). Therefore, GT prioritizes the traditional hermeneutic when conflicts arise between science and Scripture. In other words, although we acknowledge the concept of the “Two Books,” GT maintains that Scripture holds greater significance than nature. This approach enhances researchers’ understanding of both realms. It equips them to engage thoughtfully and critically with those outside the faith, thereby bridging the gap between scientific inquiry and theological reflection.

In GT, insights from behavioral and social sciences serve as valuable data sources that inform our understanding of God and His creation. Scripture provides foundational truths and principles that reveal Divine character, purpose, and moral guidance. By integrating scientific discoveries and observations about the natural world, researchers gain a broader perspective on how these elements resonate with and illuminate biblical teachings. This multidisciplinary approach allows us to appreciate the intricacies of creation while recognizing the Creator’s hand in every facet of life, from the complexities of human behavior to the patterns observed in social dynamics.

However, it is crucial to maintain a hierarchical approach in which Scripture holds ultimate authority. When researchers interpret natural phenomena and scientific findings, biblical Revelation shapes our lens. In essence, we look to Scripture to provide the interpretive framework for understanding creation rather than attempting to fit Scripture into the confines of scientific observation. By giving precedence to biblical truths, we ensure that our theological theories remain rooted in Divine Revelation, guiding our understanding of the world and its myriad complexities in ways that honor God’s Word and His Creation. This balanced perspective fosters a holistic view of faith that harmonizes spiritual understanding with the realities and scientific laws of the natural world.

While GT researchers strive to interpret natural phenomena through the lens of Scripture, it is essential to acknowledge the influence of science on biblical interpretation, as seen in how renowned scholars such as Bruce Waltke (2016) engage with the creation account in Genesis 1:3–5 (Waltke and Fredricks, 2016).

And God said, “Let there be light,” and there was light.

God saw that the light was good, and he separated the light from the darkness.

God called the light “day,” [*yôm*] and the darkness he called “night.” And there was evening, and there was morning—the first day.

In his interpretation of the Hebrew word *yôm*, translated as “day,” Waltke acknowledges several possible interpretations of the creation days, including literal 24-h periods, extended ages or epochs, and a literary framework designed to illustrate the orderly nature of God’s creation (Waltke and Fredricks, 2016). These views reflect the tension between theological and scientific concerns in interpreting the Genesis creation narrative. While some readers and scholars support a literal interpretation of *yôm* as 24-h days, this view presents challenges compared to modern scientific understanding, as most scientists reject the idea of a young Earth and of creation occurring in literal 24-h periods (Keathley et al., 2017). Walke writes, “In the case of the first suggestion [literal 24-hour periods], most scientists reject a literal 24-hour period.” (Waltke and Fredricks, 2016).

Given these scientific challenges, Waltke ultimately concludes that the literary framework interpretation is most consistent with the text’s theological emphasis. He argues that the primary focus of Genesis 1 is not to provide a scientific account of creation but to convey theological truths about God’s sovereign and orderly creation. Waltke suggests that the creation account is not bound to scientific precision but is instead structured to communicate the majesty of the Creator and the covenant relationship between God and His people. This approach enables a more flexible reading of texts, aligning the interpretation with both ancient theological concerns and modern scientific knowledge. By acknowledging multiple interpretations of the creation account, Waltke demonstrates that GT researchers must carefully approach biblical interpretation, prayerfully relying on the Holy Spirit, and consider scientific insights without compromising the theological integrity of Scripture.

## Scripture as primary data

In GT, Scripture passages can be unstructured (individual words and phrases), semi-structured (sentences), or structured (narratives, speeches, stories, parables, and sermons). This framework enables researchers to delve into the richness of theological themes and insights, facilitating an open-ended exploration of their perceptions and understandings of biblical concepts. In GT, the narrative passages of Scripture serve as the framework for unstructured interviews in traditional grounded theory. This approach enhances the examination of theological concepts such as resilience, hope, forgiveness, faith, justice, morality, and ethics. It ensures that the findings remain deeply rooted in Scripture, providing a robust foundation for academic inquiry and practical application in ministry.

A distinctive feature of grounded theory is “constant comparisons,” which serve as built-in checks and balances on the data collected during interviews (*Grounded theology constant comparisons are developed from Strauss, Anselm and Corbin, Juliet*, 1990). In GT, constant comparative analysis plays a crucial role by enabling the ongoing refinement of emerging theological theories. This process involves cross-referencing emerging theories with existing scholarly literature and Church doctrine, ensuring that new insights are grounded in established knowledge and understanding. Furthermore, this comparative analysis can extend beyond traditional biblical and theological sources to include the concept of “Two Books,” which incorporates insights from the sciences, such as social sciences and behavioral health.

GT’s holistic approach enables a deeper exploration of how theological principles align with contemporary insights, fostering a more integrated understanding of faith that encompasses both spiritual and empirical dimensions. This process ensures that the researcher’s interpretations remain consistent with established Christian doctrinal principles, enriches the study by integrating scholarly insights, and strengthens the rigor of the research, ultimately contributing to a more nuanced understanding and description of the theological theory.

## Codifying scripture and creation

### Illumination coding: fracturing scripture for initial themes

The Illumination Coding phase in GT involves selecting Scripture passages to identify fundamental themes and recurring theological patterns. In grounded theory, Birks and Mills (2015) described this process as “fracturing the data,” (Birks and Mills, 2015) which allows researchers to compare words, narratives, and theological motifs across various texts, facilitating the emergence of initial codes and broader categories (Birks and Mills, 2015). In GT, this stage involves meticulously analyzing key biblical terms and phrases, examining their semantic range, original languages, historical contexts, literary structures, and theological significance across the biblical canon. This analytical approach often reveals subtle nuances and recurrent motifs woven throughout Scripture, ensuring that interpretations remain faithful to the text. In the Illumination Coding phase, empirical evidence from Creation is examined for fundamental patterns and recurring motifs, such as observable laws, cycles, and structures in nature.

### Theological sampling

Theological Sampling plays a crucial role in refining the coding process. Instead of selecting passages arbitrarily, GT researchers must rely on biblical cross-referencing, hermeneutical comparisons, and literary parallels to enhance the theological coherence of their study. Established interpretative techniques, including analyzing structural devices, identifying figures of speech, and examining metaphorical language, will ensure that emerging theological insights are rooted in a faithful exegesis of Scripture rather than subjective presuppositions (Kostenberger and Patterson, 2019). This stage will illuminate deeper dimensions of biblical texts and prepare the foundation for subsequent coding phases. Theological Sampling also involves selecting and cross-referencing empirical data, including scientific findings, historical events, and experiential phenomena, to clarify emerging themes and deepen the coherence of the developing theory.

### Harmonization coding: abstracting theological categories

Once key themes and patterns are established, GT researchers proceed to the Harmonization Coding phase, in which foundational insights from Illumination Coding are integrated into broader conceptual theological categories. As theological categories take shape, broader biblical narratives are examined to test the consistency and interrelation of emerging theological themes. During this coding stage, insights from empirical evidence are integrated into broader conceptual categories, and their consistency and interrelation with theological constructs derived from Scripture are tested.

### Synthesis coding: refining theological concepts

At the Synthesis Coding stage, empirical and theological categories are refined into highly abstract yet deeply interconnected theological principles. This phase requires GT researchers to document the decision-making process through detailed journaling, ensuring that theological constructs remain faithful to Special Revelation while systematically grounded in the coding process. Journaling should record decisions about data collection, category refinement, theological sensitivity, and the interrelationships among higher-level concepts. This iterative process enables the development of theological theory, ensuring that final interpretations are both academically rigorous and spiritually insightful.

### Summation coding: converging theological insights

Summation Coding is the culmination of the GT coding process, where empirical and theological insights converge into their most essential, functional, and applicable form. Just as Jesus “summed up” the Law and the Prophets in Matthew 7:12, distilling their entirety into the principle of treating others as one would wish to be treated, this coding phase integrates the theological constructs refined through prior coding stages into a cohesive, actionable framework. Researchers formally articulate their theory at this stage, ensuring that the insights developed through Illumination, Harmonization, and Synthesis Coding are structured, well-defined, and ready for scholarly presentation and practical application. This coding stage converges empirical findings into a unified framework, demonstrating how the patterns and principles observed in Creation complement and illuminate theological understanding. Ultimately, Summation Coding transforms theological discovery into a fully developed theological theory, whether as a model, framework, or other construct, providing the final synthesis for academic discourse, ministry application, and functional theology.

1) *From illumination to harmonization*: Illumination Coding identifies patterns and insights from both Special and General Revelation, uncovering theological truths. This initial phase gathers raw data from Scripture and observable reality, providing the foundation for discovery.
2) *From harmonization to synthesis*: Harmonization Coding ensures that theological patterns align across different sources, reconciling biblical exegesis with empirical and historical insights. It acts as a bridge between discovery and integration.
3) *From synthesis to summation*: Synthesis Coding assembles harmonized patterns into cohesive theological constructs, allowing for structured doctrinal formation. However, without Summation Coding, these constructs remain complex and multi-layered.
4) *Summation as the final stage*: Summation Coding distills and clarifies previous stages of coding, making it functional, communicable, and applicable. Summation coding is the phase where theological complexity is transformed into accessible, actionable wisdom, just as Jesus distilled hundreds of Old Testament laws into a single principle of love and reciprocity.

By following this structured coding framework, GT researchers ensure that theological insights emerge from Scripture and are informed by methodological rigor aligned with the principles of qualitative research. The coding process in GT mirrors how Divine truth unfolds in Scripture: progressively revealed, interconnected, and illuminated by the Holy Spirit (Tuck, n.d.; Table 2).^1^

**Table 2:** Grounded theology coding phases.

Coding phase	Process in GT	Example from dual revelation
Illumination coding	Identifying initial theological themes from Scripture and empirical observation.	Observing the regularity of natural cycles, such as the changing seasons, as evidence of order in creation.
Harmonization coding	Categorizing themes into broader theological frameworks.	Connecting environmental stewardship practices with biblical mandates to care for creation.
Synthesis coding	Synthesizing findings into theological theories.	Synthesizing patterns of restoration in ecosystems with the biblical theme of renewal and reconciliation.
Summation coding	Distilling and articulating the developed theological theory into a structured, applicable framework.	Summing up the harmony between scientific laws and biblical affirmations of God’s wisdom and design.

## Methodological rigor and quality

The careful and systematic use of key GT methods sharpens the analysis and leads to the development of an integrated, comprehensive theological theory that explains a particular process or phenomenon. The outcome of a GT study is presented as a set of interconnected concepts that form a cohesive whole, yielding a substantive theological interpretation or explanation of the issue under study (Amundson, 2026). The defining feature of GT is that its theories are derived directly from data collected and analyzed by the researcher, rather than imposed from outside sources (Amundson, 2026). Accordingly, to ensure high-quality research, it is essential to maintain quality and rigor throughout the entire process. Quality and rigor in GT depend on three main factors: the researcher’s expertise and skills, the alignment between the research question and the chosen methodology (methodological congruence), and the careful, precise use of GT research methods (procedural precision) (Birks and Mills, 2015).

GT also aims to mitigate cognitive biases in theological research. Cognitive biases are mental shortcuts that influence how individuals interpret information, often leading to errors in judgment and decision-making (Kahneman, 2011). These biases can significantly impact theological study by reinforcing preconceived notions rather than allowing for an unbiased understanding of Scripture and experience. In theological inquiry, cognitive biases may manifest in several ways. The following biases, adapted from Kahneman’s (2011) foundational work and further explored in biblical studies by Chalmers (2016), include confirmation bias, the availability heuristic, overgeneralization, and anchoring bias (Kahneman, 2011). Each of these can distort the process of biblical interpretation and theological reflection.

GT actively mitigates cognitive biases through a range of methodological safeguards. Researchers are required to practice reflexivity by systematically documenting their assumptions, theological commitments, and interpretive decisions throughout the research process. The GT framework employs triangulation by cross-referencing emerging theological insights with empirical findings, established doctrinal sources, and relevant academic literature, ensuring a balanced and comprehensive interpretation. The structured GT coding sequence encourages researchers to revisit and refine their interpretations at each stage, which helps reduce the risk of anchoring or overgeneralization. Additionally, GT guards against theological relativism by maintaining that theological truths are grounded in Scripture and critically examined for validity. By integrating these practices, GT enables researchers to recognize and counteract cognitive biases to ensure that theological conclusions are thoroughly and objectively evaluated.

GT further strengthens methodological rigor by encouraging researchers to actively seek out and document evidence that challenges or contradicts emerging theological theories. This commitment to engaging with disconfirming evidence ensures that theological conclusions are not only supported by confirming data but are also tested against alternative interpretations and potential counterexamples. Such a practice enhances the validity and robustness of the findings, promoting a more balanced and critical approach to theological inquiry.

Importantly, the GT coding process is intentionally designed to be transparent and reproducible. Each stage of analysis is clearly documented and structured, enabling other researchers to follow the same methodological steps and arrive at comparable conclusions, regardless of their confessional background. While the interpretive process in GT is guided by the Holy Spirit and shaped by the researcher’s theological commitments, the coding procedures and documentation are designed to be transparent and reproducible, fostering scholarly dialogue and critical evaluation across diverse research contexts.

## Grounded theology in action

The following worked example is intended to illustrate the practical application of the GT coding process in a real-world research context. While this example demonstrates the systematic steps and potential insights yielded by GT, it is not presented as a formal validation of the method itself. Rather, it aims to show how GT can be implemented by researchers and practitioners, with the understanding that further studies may be needed to evaluate the method’s comparative effectiveness or generalizability.

The worked example draws on qualitative data collected from the author’s doctoral studies at Regent University (Amundson, 2025). The study employed a mixed methods approach, focusing on a sample of highly trained and experienced Special Weapons and Tactics (SWAT) and Emergency Response Team (ERT) officers from across the United States. These officers, selected for their extensive training and peak physical and psychological health, represent a critical segment of modern law enforcement.

Purposeful sampling was used to identify participants: the top 50 SWAT officers out of 188 who completed the National Tactical Officers Association (NTOA) 2024 Physical Fitness Qualification (PFQ) Test and submitted their official scorecards were invited to participate in the survey. Of these, 40 officers completed the survey (*N* = 40). Officers responded to the open-ended narrative question, “Please describe what resilience means to you.” These narrative responses provide qualitative data for the GT worked example.

### Research problem

The qualitative phase examined how elite tactical officers understand and experience resilience in the context of their high-risk, high-responsibility duties. The guiding research question for this example is: How do SWAT and ERT officers describe and conceptualize resilience?

### Philosophical assumptions

To explore this question, the study adopts a phenomenological perspective, assuming that the subjective experiences and personal interpretations of SWAT and ERT officers are central to understanding resilience in tactical law enforcement contexts. Their own language and meaning-making processes are treated as primary empirical data.

### Methodological approach

Given this focus on lived experience and meaning, the study employs a qualitative methodological approach. The aim in this phase is not to measure resilience quantitatively but to explore how officers define it, what dimensions they emphasize, and how they link resilience to their work, relationships, and spiritual lives.

### Research design and methods

Within the broader mixed-methods design, this qualitative component uses an online survey that includes both quantitative measures (Likert-scale items) and open-ended questions. The key qualitative item for this worked example asks officers to describe what resilience means to them in their own words. This format allows participants to articulate their understanding of resilience without being constrained by predefined categories.

### Data collection and GT coding

Narrative responses to the resilience question were collected electronically and exported for qualitative analysis. GT was then applied to these responses using the four coding phases described earlier.

1) *Illumination coding*: In the Illumination Coding phase, the researcher fractured the narrative data into discrete units of meaning, identifying specific, recurring themes in officers’ descriptions of resilience. Codes included bounce back, physical fitness, grit, adaptation, recovery, selflessness, brain health, perseverance, positive outlook, and faith in Christ, as well as references to performing under stress, growing through adversity, and being present for family despite job demands.
2) *Harmonization coding*: During Harmonization Coding, these empirical codes were integrated into broader theological categories by bringing them into dialogue with Scripture. Themes such as recovery and adaptation, strength through adversity, holistic resilience (mental, physical, spiritual, social), selfless service, learned skill, and disciplined mindset were harmonized with passages such as Ephesians 6 (spiritual armor), Romans 12:2 (renewing the mind), Matthew 7:12 (selflessness), Proverbs 6:6–8 (diligence), and Philippians 4:13, James 1:2–4, and Romans 5:3–4 (strength through Christ, perseverance through trials, suffering producing hope).
3) *Synthesis coding*: In the Synthesis Coding phase, these harmonized categories were refined into more abstract theological constructs. Resilience emerged as a reality cultivated through faith, discipline, and community; as a process of adaptation, recovery, and growth; and as something grounded in purpose and selflessness. These constructs were supported by both Scripture (for example, James 1:2–4 on joy in trials, Romans 12:2 on transformation, Philippians 3:14 on pressing toward the goal) and the empirical literature on positive psychology and resilience-building practices.
4) *Summation coding*: Finally, Summation Coding distilled these constructs into a functional theological theory of resilience for law enforcement. In this framework, resilience is understood as Spirit-enabled adaptation, recovery, and growth through adversity. The process is holistically integrated across spiritual, physical, mental, and social dimensions and is strengthened by faith, disciplined practice, community, and selfless service. Foundational biblical texts such as Philippians 4:13, Ephesians 6, and Romans 5:3–4 affirm faith as the ultimate source of strength.

This worked example demonstrates how GT moves from raw qualitative data drawn from SWAT and ERT officers’ lived experience to a coherent, applicable theological theory of resilience that integrates the “Two Books” of Special Revelation (Scripture) and General Revelation (Creation). The example also illustrates the systematic coding sequence of GT and its distinct advantages over other qualitative theological approaches. Unlike narrative inquiry or ethnographic methods, which often focus solely on personal experience or social context, GT integrates empirical findings with theological reflection, ensuring that interpretations are grounded in both lived experience and scriptural authority. The harmonization and synthesis phases of GT move beyond descriptive analysis, allowing researchers to test and refine emerging themes against biblical texts and established doctrine. This process yields theological insights that are both contextually relevant and doctrinally robust.

**Figure 3:**
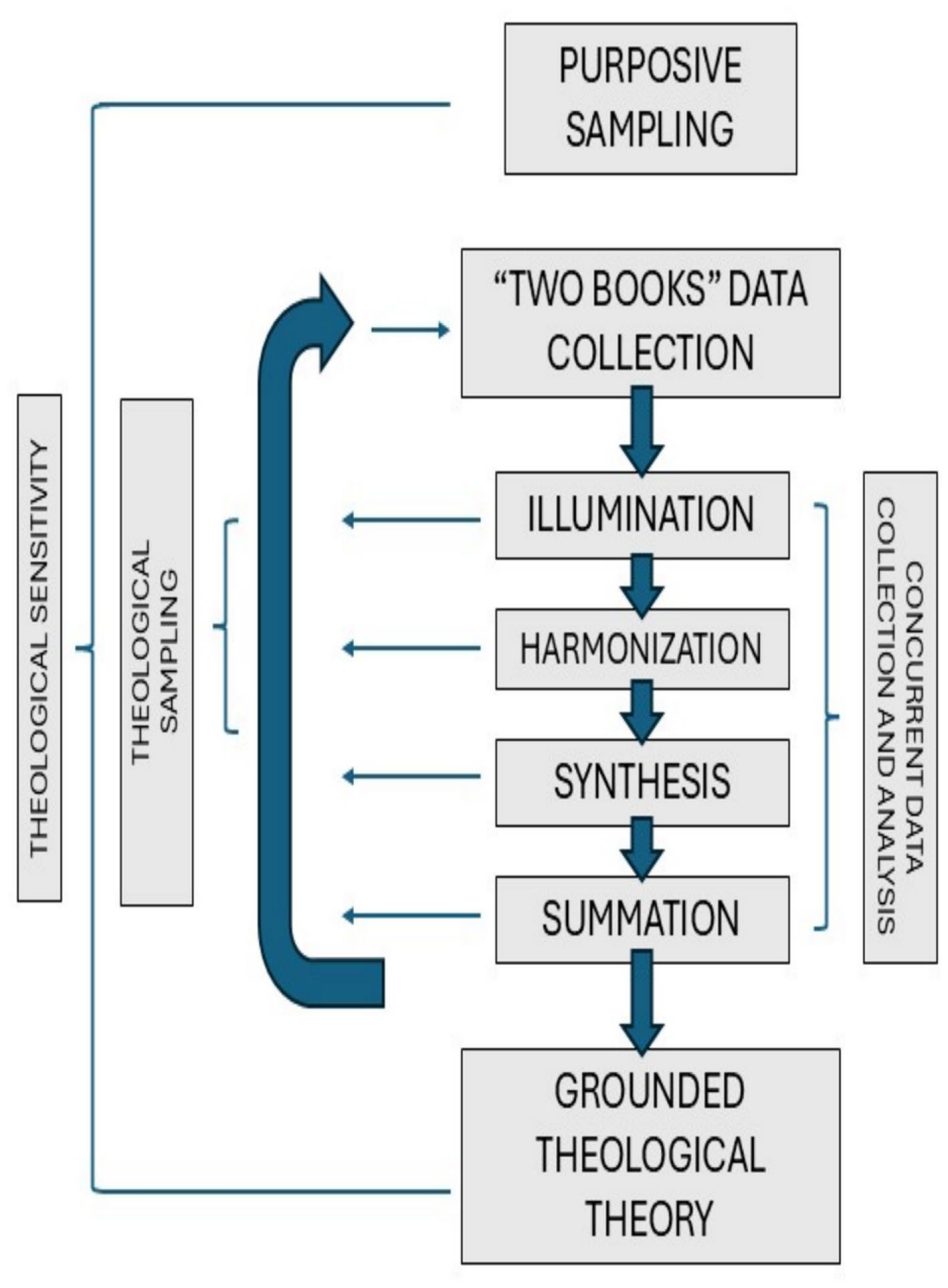
Grounded theology design framework.

For D. Min. students and Christian researchers, the worked example provides a reproducible framework that bridges empirical research and theological interpretation. It enables practitioners to draw on years of theological and biblical study while also engaging with sociological research methods. The result is a holistic, Spirit-led understanding of resilience that is applicable to real-world ministry and professional practice. By explicitly connecting empirical data to scriptural and doctrinal foundations, GT offers a methodological pathway for developing practical theology that is both academically rigorous and spiritually transformative (Figure 3; Table 3).

**Table 3:** Worked example of GT coding process.

Coding phase	Process in GT	Codes (“two books” approach)
Illumination coding	Fracture officers’ narrative responses to identify specific, recurring empirical themes from lived experience.	Bounce back, physical fitness, grit, adaptation, recovery, selflessness, brain health, perseverance, positive outlook, faith in Christ, ability to perform under stress, ability to grow through adversity, ability to be present for family despite job stress, learned skill, trained and mentored resilience
Harmonization coding	Harmonize empirical codes with Scripture and integrate them into broader theological categories.	Recovery and adaptation, strength through adversity, holistic resilience (mental, physical, spiritual, social), selfless service, learned and cultivated skill, positive and disciplined mindset; Ephesians 6 (spiritual armor), Romans 12:2 (renewing the mind), Matthew 7:12 (selflessness), Proverbs 6:6–8 (diligence), Philippians 4:13 (strength through Christ), James 1:2–4 (perseverance through trials), Romans 5:3–4 (suffering produces hope)
Synthesis coding	Refine harmonized categories into abstract theological constructs that integrate empirical patterns and biblical themes.	Resilience cultivated through faith, discipline, and community; resilience as adaptation, recovery, and growth; resilience grounded in purpose and selflessness; James 1:2–4 (joy in trials), Romans 12:2 (transformation), Philippians 3:14 (pressing toward the goal); empirical support from positive psychology and resilience-building practices
Summation coding	Distill theological constructs into a functional, applicable theological framework.	Resilience is understood as Spirit-enabled adaptation, recovery, and growth through adversity. The process is holistically integrated across spiritual, physical, mental, and social dimensions and is strengthened by faith, disciplined practice, community, and selfless service. Foundational biblical texts such as Philippians 4:13, Ephesians 6, and Romans 5:3–4 affirm faith as the ultimate source of strength.

## Conclusion

GT offers a structured, rigorous, and Spirit-led approach to theological inquiry, integrating both Special Revelation (Scripture) and General Revelation (Creation) as complementary sources of Divine knowledge. By adapting grounded theory methods, researchers can develop substantive, applicable theological frameworks that are firmly rooted in data and faithful to biblical truth. This paradigm fosters academic rigor, methodological transparency, and spiritual discernment, ensuring that theological insights are not only empirically sound but also transformative for ministry, scholarship, and engagement with the broader world. Ultimately, GT bridges the gap between faith and reason, demonstrating that disciplined qualitative research, combined with Divine guidance, illuminates the depth and relevance of the “Two Books” theory for addressing contemporary challenges.

## Data Availability

Publicly available datasets were analyzed in this study. This data can be found here: Amundson, Gregory J. “Guarding the Heart: New Constructs in Chaplaincy and Resilience Pathways in Law Enforcement.” ProQuest Dissertation and Thesis, Regent University, 2025.

## Footnotes

1 Although organizing and coding data provides an opportunity for rich analysis, proper knowledge, as outlined in the Bible, emerges through obedience and is fully realized when lived out in the community. Scripture emphasizes that understanding is not merely an intellectual exercise; it becomes meaningful when applied in relational contexts. For instance, James 1:22 reminds us to be doers of the word, not just hearers. Authentic knowledge manifests through action and community engagement, where Christians can experience and grow in understanding God’s truth together.

## Author’s note

This article draws on the conceptual framework and methodological guidance presented in Tie Chun, Ylona, Melanie Birks, and Karen Francis, “Grounded Theory Research: A Design Framework for Novice Researchers,” SAGE Open Medicine 7 (2019): 1–8.
